# Eco-friendly estimation of isosorbide dinitrate and hydralazine hydrochloride using Green Analytical Quality by Design-based UPLC Method

**DOI:** 10.1039/d1ra04843k

**Published:** 2021-09-07

**Authors:** Hemanth Kumar Chanduluru, Abimanyu Sugumaran

**Affiliations:** SRM College of Pharmacy, SRM Institute of Science and Technology Kattankulathur- 603203 India abipharmastar@gmail.com abimanys@srmist.edu.in +917904062599

## Abstract

Isosorbide dinitrate (ISD) and hydralazine hydrochloride (HDZ) are critical drugs for the treatment of heart failure. Currently, no available analytical method for the determination of ISD and HDZ exists as per the literature that combines UPLC and Green Analytical Quality by Design, which is critical for designing a method that is sustainable for long-term use. This study proposes an eco-friendly determination of isosorbide dinitrate (ISD) and hydralazine hydrochloride (HDZ) using a Green Analytical Quality by Design-based UPLC Method. The developed technique is capable of separating ISD and HDZ, as well as their degradation products, using a Phenomenex C18 (50 × 2.1 mm, 2 μm) column containing ethanol and 0.1% trifluoroacetic acid (60 : 40% v/v) at a flow rate of 0.5 mL min^−1^. This technique was validated and established a linearity range of 10–60 μg mL^−1^ and 18.75–112.5 μg mL^−1^, with *R*^2^ of 0.9998 and 0.9992 for ISD and HDZ, respectively along with accuracy, reproducibility, and selectivity. The new approach was further evaluated using five different assessment techniques such as National Environmental Methods Index, Analytical Eco-Scale, Green Analytical Procedure Index, Analytical Method Greenness Score, and Analytical GREEnness Metrics, and was determined to be environmentally benign. Based on these results, we have concluded that the developed UPLC technique with the combined approach of Green Analytical Quality by Design for determining stability might benefit in the creation of novel pharmaceutical products such as isosorbide dinitrate and hydralazine.

## Abstract 2

Analysing isosorbide dinitrate and hydralazine by using an eco-friendly method is an initial stepping stone towards environmentally benign method development, and its combination with the AQbD makes it the method to use for ages without revalidation.

## Graphical abstract

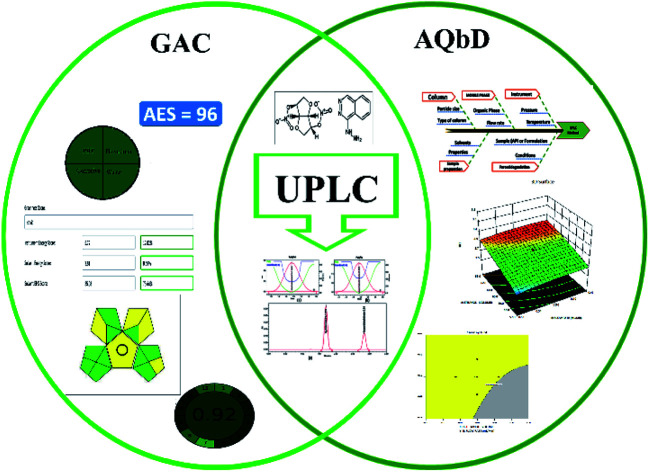

## 1. Introduction

Liquid chromatography requires solvents with a combination of organic modifiers and pH enhancers to develop a stable analytical method. The most frequently used organic modifiers are acetonitrile, methanol, *n*-hexane, and tetrahydrofuran. These solvents have substantial impact on analytical chemistry due to their advantages of compatibility with most chemical compounds and drugs with low signal-to-noise ratio but have a notorious effect on the environment and analyst.^1^ Green analytical chemistry (GAC) has mainly focused on developing analytical methods that are safe for the environment and the analyst. GAC works on 12 principles^2–6^ that mainly focus on reducing solvent usage, replacing toxic chemicals, reusing the generated waste, and avoiding unnecessary steps.

Hence, there is a need to establish the analytical method based on GAC principles. However, liquid chromatographic methods may follow 11 out of 12 principles.^7^ The reduction of solvent usage may be achieved with the help of miniaturization. The use of UPLC instead of HPLC reduces the waste generated and the time of analysis due to high pressure and low particle-sized short columns. This is a positive effect over the generation of low waste by UPLC technique within a quick run time. Replacement of more hazardous solvents with the help of eco-friendly solvents needs to be considered by the analysts. Selection of suitable eco-friendly solvents could be achieved with the help of several solvent selection guidelines proposed by different companies, such as Sanofi's Solvent Selection Guide,^8^ GSK Solvent Selection Guide,^9^ and SmithKline Beecham Solvent Selection.^10^ Most of the guidelines categorize the solvents based on environmental hazard safety (EHS) and net cumulative energy demand (CED).

Very few solvents were available as eco-friendly with low CED values, but these solvents cannot be used directly as an organic modifier in the analysis of drugs due to their disadvantages such as high noise in the instrument, and poor compatibility with all the drugs. By considering this fact, we have considered that ethanol was a green solvent, which is the perfect alternative for hazardous methanol and propylene carbonate for acetonitrile.

Application of Analytical Quality by Design (AQbD) in analytical methods^11^ helps to enhance the principles of GAC. The use of AQbD in the development of a method has many advantages: (i) it saves time and waste, (ii) revalidation is not required, (iii) the method can be transferred directly to other systems, and (iv) regulatory compliances.

Combining these three principles is innovative and essential in developing a stable method for long-term usage. Application of AQbD nullifies revalidation and enhances GAC principles. UPLC helps in reducing waste generation and supports GAC principles. Finally, the AQbD-based method in UPLC is always a robust method. Hence, this tri-combination is considered complementary to each other, and enhances the importance of analytical development.

**Fig. 1:**
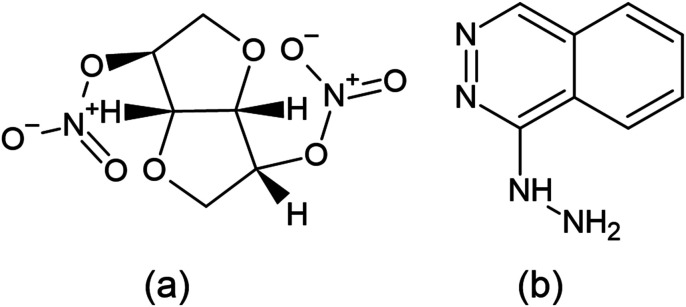
Structure of isosorbide dinitrate (a) and hydralazine (b).

Isosorbide dinitrate (ISD) is chemically (3*R*,3*aS*,6*S*,6*aS*)-hexa hydrofuro-[3,2-*b*]-furan-3,6-diyldinitrate^12^ (Fig. 1), which is soluble in alcohols and sparingly soluble in water. Guanylate cyclase is an essential enzyme activated by nitric oxide once ISD reaches the systemic circulation. This guanylate cyclase enzyme elevates the concentration of cyclic guanosine-3′,5′-monophosphate (cGMP), which activates protein kinases and triggers a sequence of phosphorylation feedbacks, leading to dephosphorylation of smooth muscle fiber myosin light chains. Eventually, calcium ions are released and induce vasodilation, and cause smooth muscle relaxation. It mainly targets and antagonizes atrial and brain natriuretic peptide receptors, which are effective vasoactive hormones.

Hydralazine hydrochloride (HDZ) is chemically (1*E*)-1-hydrazono-1,2-dihydro phthalazine hydralazine^13^ (Fig. 1), and may relax the arteriolar smooth muscle and lower blood pressure by interfering with calcium transport in the vascular smooth muscle by an unknown mechanism. Calcium transport may be disrupted by preventing calcium influx into the cells, suppressing calcium release *via* intracellular compartments, acting directly on actin and myosin, or a combination of these processes. Increased stroke volume, cardiac output, and heart rate result from a decrease in the vascular resistance.

The HDZ drug comes under the Biopharmaceutical Classification System (BSC) class III with high solubility and low permeability. However, ISD also comes under class I due to its high permeability in the oral, rather than the gastrointestinal tract.

The US Food and Drug Administration has approved the combination of ISD and HDZ for the use of African American patients, in addition to standard heart failure medications.^14–16^ When related to conventional treatment alone, this mixture has been shown to advance survival, sustain the value of life, and help to reduce the risk of the first hospitalization for heart failure.

Several methods are available to analyze these drugs individually and in combined dosage form using UV,^17^ HPLC,^18–22^ and LCMS.^23^ However, so far, no single method was developed using GAC principles or using a robust AQbD^24^-based UPLC method.

Furthermore, no article has been published to explore the ISD and HDZ using this triple combination (GAC, AQbD, and UPLC). This study aimed to develop a simple, sensitive, fast, reliable, environmentally friendly, and specific UPLC method for determining ISD and HDZ simultaneously. The method validation is carried out under the ICH Q2 guidelines (R1).

## 2. Experimental

### 2.1. Chemicals and reagents

ISD and HDZ were obtained as a gift sample from Glenmark formulations (Mumbai, India). Union pharmaceuticals and chemicals (Mumbai, India) supplied HPLC grade organic phase ethanol. Merck (Mumbai, India) supplied analytical grade trifluoracetic acid, hydrochloric acid (HCl), sodium hydroxide (NaOH), orthophosphoric acid, and 30% hydrogen peroxide. Purified water for HPLC was produced using a Milli-Q filter system.

### 2.2. Equipment and software

The development of the liquid chromatography (LC) analytical technique was carried out using an Agilent UPLC (Santa Clara, California, USA) integrated with a binary solvent controller, an automated sampler, a photodiode array detector (PDA), and temperature-controlled column compartments. The pH of the solutions was determined using a Systronics Digital pH Meter 802 (Gujarat-India). Unichrome ultrasonic baths were utilized to degas the solvents. Phenomenex (C18, 50 × 2.1 mm, 2 μm) (Phenomenex, Torrance, CA, USA) columns were used for trails to determine ISD and HDZ. Agilent LC systems were integrated with the chromatography data management software Empower 3. AQbD was carried out using Design-Expert® trailed version 12 (Stat-Ease Inc., Minneapolis-USA).

### 2.3. Final conditions for the UPLC method

The final proposed technique constraints within the working point were Phenomenex C18 (50 × 2.1 mm, 2 μm) column; mobile phase A (0.1% trifluoracetic acid); B (HPLC grade ethanol); strong needle wash = HPLC grade ethanol; 0.5 mL min^−1^ used as the rate of pump flow; injection volume 5 μL; column temperature 35 °C ± 1; detection wavelength 270 nm.

### 2.4. Preparation

#### 2.4.1. Preparation of 0.1% trifluoroacetic acid

Trifluoracetic acid (TFA) (1 mL) was transferred through a dry clean positive-displacement pipette to a 1000 mL container containing water and made up to the final volume.

#### 2.4.2. Method development and sampling procedures for the AQbD study

The ISD and HDZ stock solutions were prepared with ethanol at concentrations of 40 and 75 μg mL^−1^, respectively. One mL of the prepared stock solutions was transferred into a 10 mL volumetric flask and filled with ethanol. The working sample solution was filtered into a vial using a 0.22 μm polyvinylidene fluoride (PVDF) filter.

#### 2.4.3. Validation samples for the UPLC method

All validating samples were produced with the UPLC sample solution (ethanol and 0.1% TFA 60 : 40 v/v). The purity of the ISD and HDZ drug substances utilized was 98.0 to 102% for both drugs. Three replicates of a sample solution containing a defined concentration of ISD and HDZ were produced to determine the linearity. They were diluted to the concentrations of about 10, 20, 30, 40, 50, and 60 μg mL^−1^ for ISD and 18.75, 37.5, 56.25, 75, 93.75, and 112.5 μg mL^−1^ for HDZ. A concentration range (between 40 and 75 μg mL^−1^) was chosen based on the marketed formulation within the range, and solutions for accuracy and precision were developed (80, 100, and 120% target concentration). The accuracy and repeatability accessed using the solutions derived from linearity were applied (three replicates).

## 3. Results and discussion

Developing an analytical method by keeping intact GAC principles was a revolutionary ideology for a sustainable, eco-friendly method. However, the development of a green analytical method without applying the AQbD approach may suffer from method performances and needs revalidation. Application of these principles together in the UPLC method helps to enhance the method stability, along with sustainability. So, we have combined these three combinations of approaches to develop an eco-friendly and robust method. The whole process of developing this innovative method is as follows.

### 3.1. Selection of solvent

During the method development phase, the preparation of samples has proved to be a significant stumbling block. ISD and HDZ are soluble in a wide variety of solvents often used in reverse phase liquid chromatography (RPLC) sample preparation, including methanol (MeOH), acetone, ether, chloroform acetonitrile (ACN), and water. However, employing these hazardous solvents leads to environmental complexities and impairs the principles of green analytical chemistry, so the solvent used in the approach was limited to the less hazardous organic solvent ethanol. The initial attempt made to dissolve ISD and HDZ with 100% ethanol as a solvent showed a complete solubility of the two drugs, and we intended to optimize the water content of the solvent to decrease organic solvent wastage, which is another GAC principle. 50% ethanol was utilized as a solvent, but failed to solubilize these drugs. However, ISD and HDZ dissolved well when we added 70% ethanol to the flask, even at high concentrations of 4 mg mL^−1^. Subsequently, certain solubility issues were observed with 70% ethanol as a solvent. After a week at ambient temperature, crystals began to form. Additional solvent testing was conducted using ethanol and buffer (the same one used in UPLC), rather than water. Initially, ethanol-0.1% TFA (40 : 60, v/v) was employed as a mobile phase, but this was altered to ethanol-0.1% TFA (60 : 40, v/v) due to observation of noise in the chromatogram.

### 3.2. Method scouting

Even though there are few existing HPLC^17,18,20,21^ methods for ISD and HDZ as in the literature, they frequently fail for method adoption due to the need for continuous revalidation, and thus are not environmentally sustainable due to the usage of MeOH and ACN as a mobile phase. The first time, we have developed an environmentally friendly method, and conducted a few preliminary experiments to determine the viability of the method's development and its application to the evaluation of these pharmaceuticals. Different one-factor-at-a-time (OFAT) studies were used as part of the technique scouting. The RP-LC method was chosen due to the molecule's properties and structure. The method was developed with the following parameters: mobile phase A is 0.1% orthophosphoric acid (v/v); mobile phase B is ethanol (60 : 40 v/v); Kinetex phenyl hexyl (50 × 4.6 mm, 2.6 μm) column; column temperature set to 50 °C; flow rate 0.7 mL min; detection wavelength 270 nm; isocratic elution. It shows an improper peak shape, resolution with substantial fronting and also lacks reproducibility. For LC, a single ionized state is required at the set pH to avoid tailing/fronting, which may happen if the molecule shifted between a single ionized state to another type when the set mobile phase pH and substances, due to ionization affecting the substance's retention upon stationary phase.

**Fig. 2:**
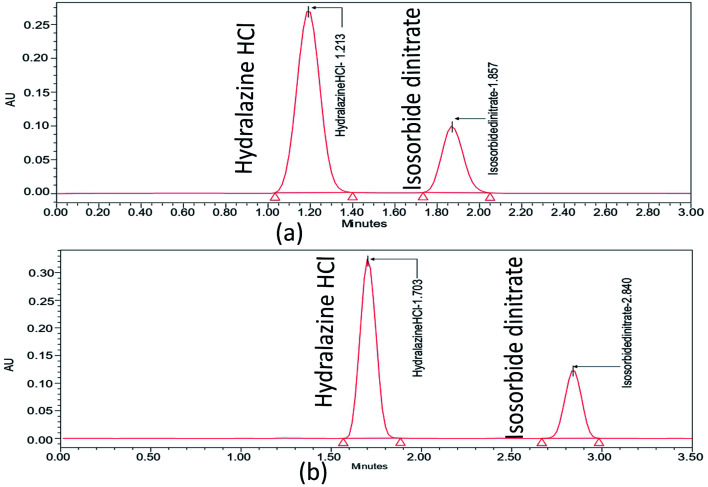
Chromatograms of ISD and HDZ in the Kinetex phenyl hexyl column with ethanol and 0.1% trifluoro acetic acid (60 : 40 v/v) (a), and Phenomenex C18 column with ethanol and 0.1% trifluoro acetic acid (60 : 40 v/v) (b).

Further choosing trifluoroacetic acid as the buffer, as part of a trial based on the literature with no pictograms and being a more environmentally friendly option, is the isocratic procedure that was expanded to ascertain the organic phase concentration in the mobile phase at which ISD and HDZ elute. The ISD and HDZ substances' peak shapes were significantly enhanced. Additionally, the degradation peaks were successfully isolated from the ISD and HDZ peaks. At this point, a stationary phase, Phenomenex C18 (50 × 2.1 mm, 2 μm), was also tested with a smaller particle size. It was chosen to continue analyzing the separation of ISD and HDZ from the previous one (Fig. 2). It produced sharp peaks and also gave a better resolution between ISD and HDZ in similar conditions. We further evaluated the method utilizing the degraded samples from the degradation trials from the ISD and HDZ drug solution, with the primary goal of increasing the selectivity between ISD and HDZ, its degradation products. Two columns were used to test the degradation samples: Kinetex phenyl hexyl and Phenomenex C18.

The form of the ISD and HDZ peaks appeared to be improved when utilizing a Phenomenex C18 column, and the separation of various degradation products appeared to be improved. Certain peaks eluted very early, with little retention, but a better ratio factor based on the properties of degraded peaks. The effect of the composition of the mobile phase was examined. Ethanol increased the peak shape of the substances and the separation of active pharmaceutical ingredients from degraded products. The pump flow and column temperature at start-up were fairly high (0.7 mL min^−1^ and 50 °C, respectively). A high temperature can significantly shorten the column's lifespan. As a result, the pumping flow rate and the temperature were reduced to increase the column lifetime (0.5 mL min^−1^ and 35 °C, respectively). Temperature changes have an effect on the optimization data. The procedure duration was increased to sufficiently elute all degradation products, but was kept at 3.5 minutes to minimize solvent wastage.

### 3.3. Identification of the analytical target profile

The Analytical Target Profile (ATP) summarizes the estimation criteria for quality attributes to meet by an analytical method. The elements of ATP for the present method were set, as determining ISD and HDZ simultaneously in the bulk and tablet dosage form using UPLC-PDA was set as a target analyte, and the stability-indicating assay method with green analytical principles was set as target method application. Finally, the UPLC approach with PDA detection was selected as the analytical technique based on ATP. The Critical Material Attributes (CMA) were chosen as the resolution, theoretical plates, and second peak retention time.

### 3.4. Preliminary risk assessment

**Fig. 3:**
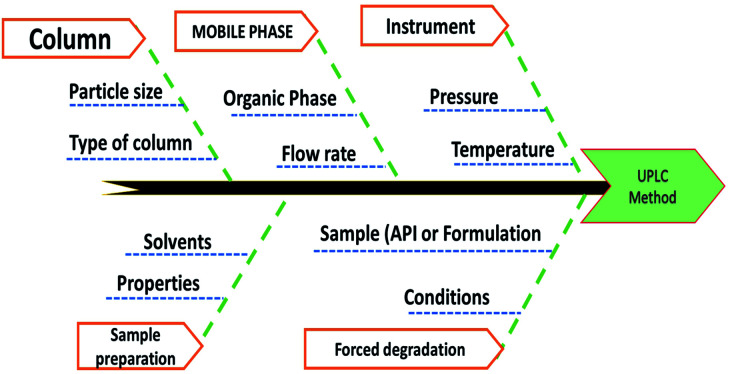
Risk assessment of the proposed method by the Ishikawa fishbone diagram.

Method risk assessment was characterized based on the “Ishikawa” fishbone diagram (Fig. 3). The following CMPs were defined as having an impact on the CMAs : organic modifier concentration in the mobile phase, sample solvent, column temperature, and mobile phase flow rate. The sample solvent was vital because an accurate determination of the drug substances depends on its total solubility warranted. Nevertheless, it was tested independently of the LC method development, resulting in the used solvent that was suitable regardless of composition variation. As a consequence, it was omitted from the AQbD procedure. Although the temperature of the column has minimal effect; nonetheless, it is not negligible. Regarding its impact on the method efficiency, it might vary significantly. We found that the knowledge about the parameter interactions would be beneficial, and thus designated the column temperature as potentially essential for further study. Generally, the pump flow is closely restricted and therefore is not considered essential. It may, however, exhibit substantial parameter interaction when combined with the percentage of organic modifiers in the mobile process. As such, it was incorporated into the CMPs that were chosen. Finally, the percentage of organic modifiers in the mobile phase, the column temperature, and the flow rate all significantly impact the method's output (CMAs) in terms of the resolutions, HDZ theoretical plates, and ISD retention time.

### 3.5. Method optimization

**Fig. 4:**
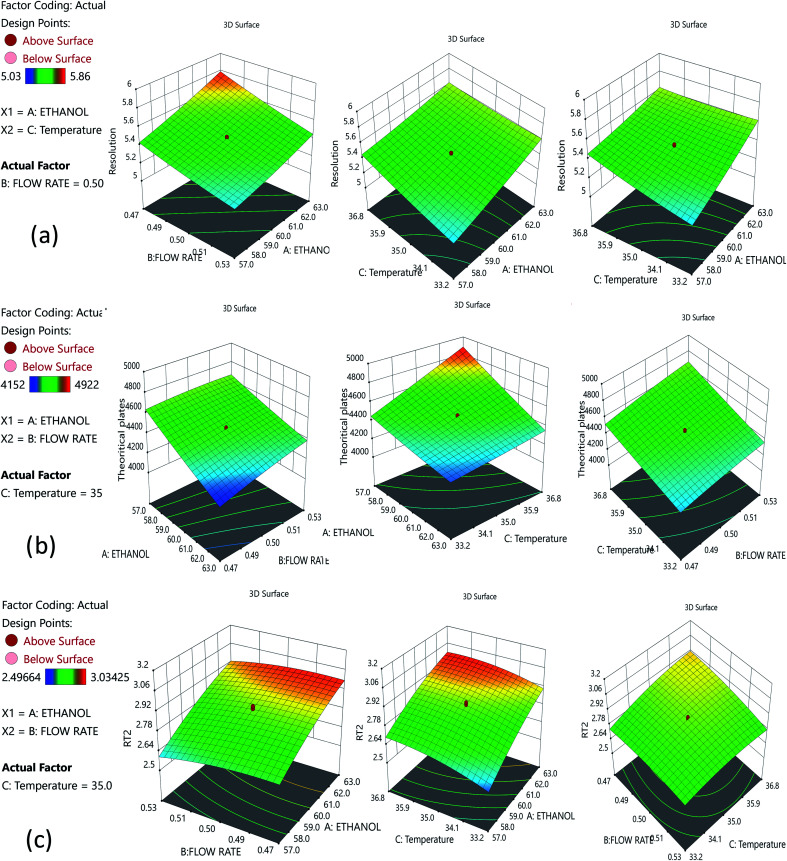
Contour plots for the optimized method: (a) interaction of ethanol and the flow rate; (b) interaction between ethanol and temperature; and (c) interaction between the flow rate and temperature.

The risk assessment method identified the optimal organic phase concentration (60%), a flow rate of 0.5 min mL^−1^, and temperature of 35.07 ± 1 °C as the most promising with Phenomenex C18 (50 × 2.1 mm, 2 μm), column; and a mobile phase composition of 0.1% TFA and ethanol (40 : 60). A reduced run time results in reduced mobile phase usage, improved laboratory time management, and waste management in adherence with GAC principles, enabling higher efficiency of analysis. Following that, we used DoE that included all of the relevant CMPs, and tracked their outcome solely on CMAs in order to analyze the required method model equations. A rotatable central composite design with a quadratic design model was used. The following parameters were chosen for the DoE analysis built on the initial method risk assessment; percentage of ethanol, column temperature (32–38 °C), and flow rate (0.45–0.55 mL min^−1^). The following parameters were chosen: resolution between two drugs >1.5; theoretical plates of the HDZ more than 3000; and retention time of the second drug. The process model equations were estimated and statistically validated using ANOVA based on the DoE results (Table 1) with each model coefficient evaluated. The model coefficients used in this portion are statistically significant (*P* values < 0.0001). Additionally, the F-ratios indicate the importance of each coefficient in the model. Higher *R*^2^ values and low lack-of-fit (LOF) values indicate a well-fitting model, whereas higher F-ratios indicate statistical significance for the analytical model equation. The contour plots for the A, B, and C interactions are depicted in Fig. 4.

**Table:** Statistical parameters of an optimized method for ISD and HDZ (ANOVA results)

Critical material attributes	Coefficient of the coded equation	*P*-Value	*F*-Value	Fit statistics
Resolution	+5.4399	<0.0001	181.59	*R* ^2^ = 0.9939
	0.15286 (A)	<0.0001	695.20	Adjusted *R*^2^ = 0.9884
	−0.14315 (B)	<0.0001	609.73	Predicted *R*^2^ = 0.9682
	0.06989 (C)	<0.0001	145.36	Std. Dev. = 0.0214
	−0.02720 (A × B)	0.0049	12.90	Mean = 5.47
	−0.06672 (A × C)	<0.0001	77.59	C.V.% = 0.3916
	0.06577 (B × C)	<0.0001	75.40	Lack of fit
	0.01602 (A^2^)	0.0176	8.06	*P* = 0.3211
	0.01248 (B^2^)	0.0514	4.89	*F* = 1.55
	0.01659 (C^2^)	0.0148	8.64	
Theoretical plates for HDZ	4472.3271	<0.0001	441.32	*R* ^2^ = 0.9975
	−183.4680 (A)	<0.0001	2180.97	Adjusted *R*^2^ = 0.9952
	59.01978 (B)	<0.0001	225.70	Predicted *R*^2^ = 0.9877
	135.7003 (C)	<0.0001	1193.14	Std. Dev. = 14.52
	42.94543 (A × B)	<0.0001	70.00	Mean = 4472.56
	−84.0680 (A × C)	<0.0001	268.24	C.V.% = 0.3246
	18.2892 (B × C)	0.0052	12.70	Lack of fit
	9.93163 (A^2^)	0.0266	6.74	*P* = 0.4068
	−12.988 (B^2^)	0.0068	11.53	*F* = 1.25
	3.40244 (C^2^)	0.3945	0.7915	
The retention time of ISD	2.8565	<0.0001	354.75	*R* ^2^ = 0.9969
	0.1589 (A)	<0.0001	2461.24	Adjusted *R*^2^ = 0.9941
	−0.0348 (B)	<0.0001	118.67	Predicted *R*^2^ = 0.9946
	0.05409 (C)	<0.0001	285.15	Std. Dev. = 0.0118
	0.00609 (A × B)	0.1758	2.12	Mean = 2.79
	−0.01127 (A × C)	0.0225	7.26	C.V.% = 0.4236
	−0.03642 (B × C)	<0.0001	75.74	Lack of fit
	−0.03183 (A^2^)	<0.0001	104.21	*P* = 0.9973
	−0.02509 (B^2^)	<0.0001	64.74	*F* = 0.051
	−0.0342 (C^2^)	<0.0001	120.30	

### 3.6. Method operable design region

**Fig. 5:**
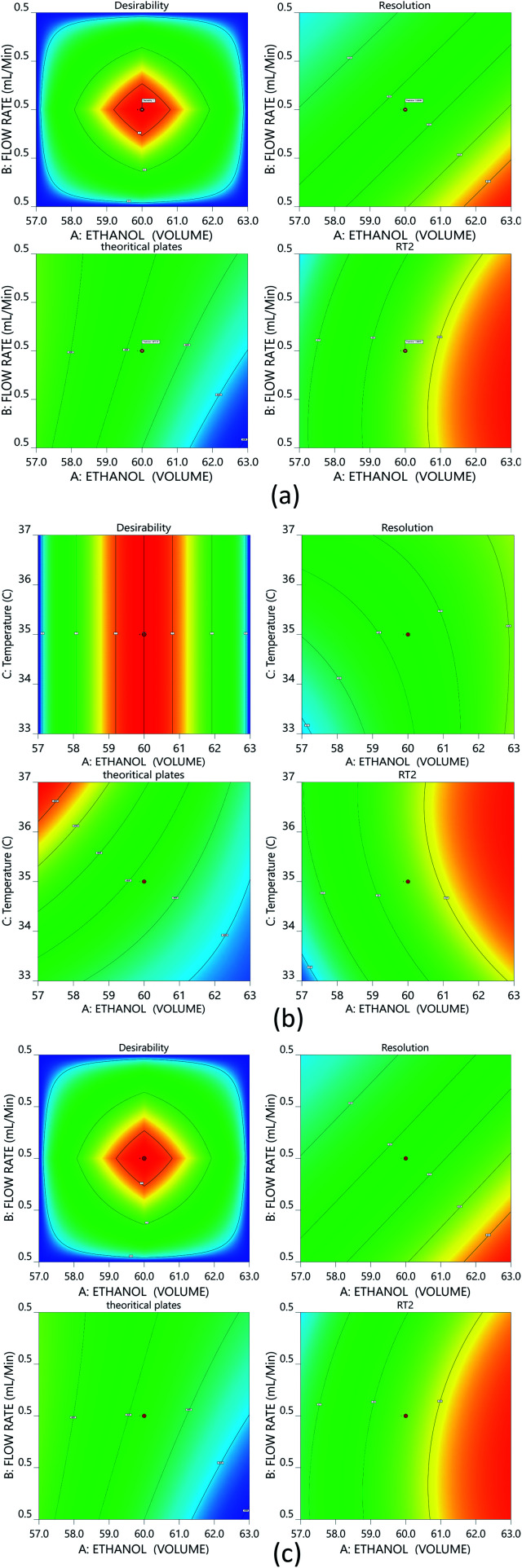
Desirability contour plots for the optimized method: (a) interaction of ethanol and the flow rate; (b) interaction between ethanol and temperature; and (c) interaction between the flow rate and temperature.

**Fig. 6:**
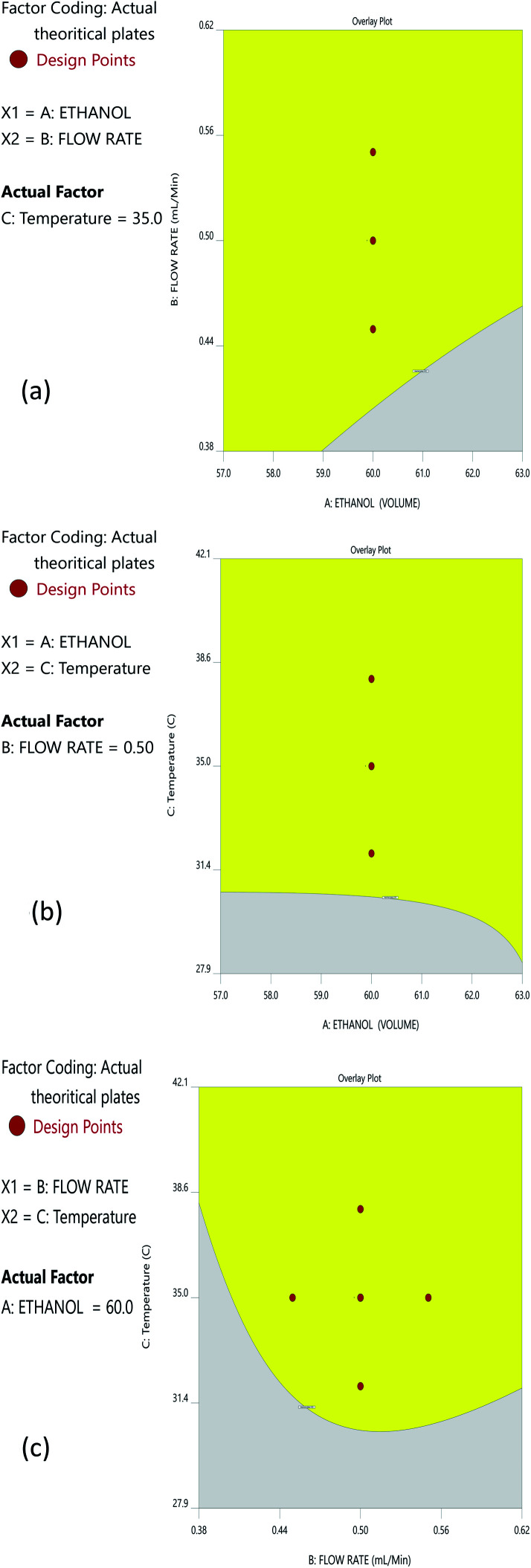
Overlay plots for the optimized method: (a) interaction of ethanol and the flow rate; (b) interaction between ethanol and temperature; and (c) interaction between the flow rate and temperature.

The Method Operable Design Region (MODR), alternatively referred to as the control space, was developed using CMA models and robustness simulations. Fig. 5 portrays the contour plots for various combinations of CMAs in the DoE region for the optimized method, which can also be called a desirability region. MODR values for robustness are as follows: flow rate at 0.45 to 0.55 mL min; column temperature at 32–38 °C. The optimal variance in the mobile phase percentage is ±5%. Within MODR, an optimal working point was selected, which is as follows: flow rate is 0.5 mL min; column temperature at 34.609 °C. At the working period, the projected CMAs were Rs is 5.425, *N* is 4442, and RT2 is 2.843. At the practically executed, the real CMAs were Rs at 5.413, *N* is 4429, and RT2 at 2.84. The overlay plots for the optimized plots indicate that the yellow region is the range that does not require any revalidation further with each interaction, and is depicted in Fig. 6.

### 3.7. Method validation

The accuracy, precision, linearity, and repeatability of the developed method were tested at the chosen working point by assessing the stability of ISD and HDZ. The method was validated in accordance with the ICH Q2 (R1) guidelines. Validation for the ISD and HDZ solutions was prepared in the manner defined in Section 2.4.2.

#### 3.7.1. Specificity

**Fig. 7:**
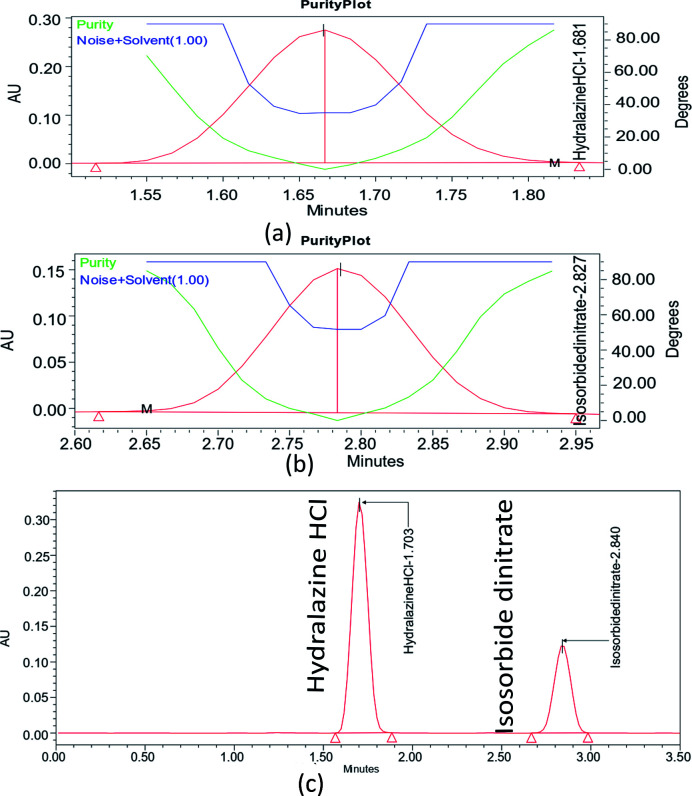
Peak purity for ISD (a), HDZ (b), and standard graph for ISD and HDZ (c).

Specificity refers to the substance's ability to be measured precisely in the presence of the matrix effect and any additives used to ensure the identification of the analyte(s) of interest. The blank solution, standard solution, and assay test solution were analyzed, and the ISD and HDZ peaks from each solution were calculated. The PDA detector was used to determine the peak purity under the specified chromatographic conditions. The purity angle must be less than the purity threshold for a peak to be considered pure. As shown in Fig. 7, the ISD and HDZ peaks in the chromatogram were found to be pure. Both compounds were totally separated, and there was no evidence of analyte retention time drift.

#### 3.7.2. Linearity

Six distinct proportions of standard concentrations were prepared individually to measure the linearity range. The calibration curve was obtained as peak area *versus* the standard solution concentration. The ISD and HDZ study solutions were prepared with a concentration ranging from 10 to 60 μg mL^−1^ and 18.75 to 112.5 μg mL^−1^, respectively. The relative response factors for ISD and HDZ were calculated by preparing standard solutions at various concentrations ranging from the LOQ to 2.797 μg mL^−1^ for ISD and 1.66 μg mL^−1^ for HDZ. The results were depicted in Table 2.

**Table:** Linearity parameters for ISD and HDZ

Parameter	ISD	HDZ
Concentration (μg mL^−1^)	10 to 60	18.75 to 112.5
Regression equation	26 411.1886*x* + 442.4000	31 588.4617*x* + 602.5333
*R* ^2^	0.9998	0.9992
Sample size	6	6
LOD (μg mL^−1^)	0.923112691	0.549820152
LOQ (μg mL^−1^)	2.797311184	1.666121672

**Fig. 8:**
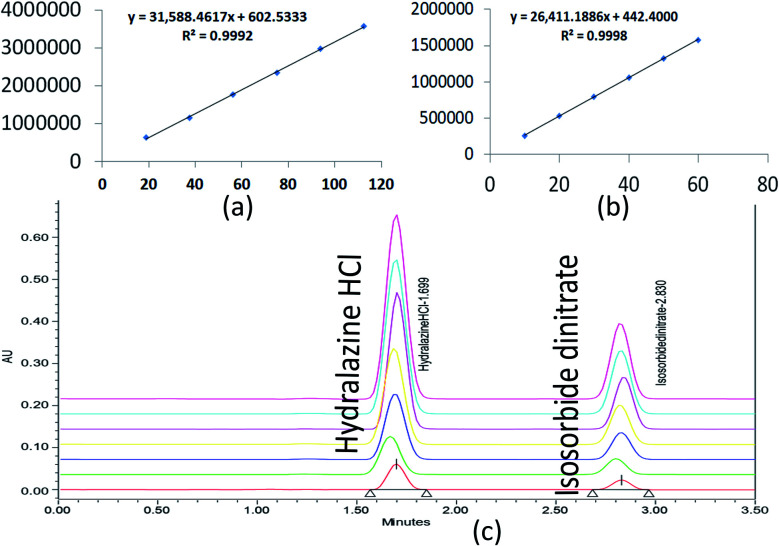
Linearity graphs for ISD (a), HDZ (b), and chromatographic overlay plot for ISD and HDZ in various linear concentrations.

The correlation coefficient (*R*^2^) should not be less than 0.998 to define the criterion of linearity. Correlation coefficients (*R*^2^) greater than 0.998 were observed in Fig. 8. As a result, the analytical procedure was linear over this concentration range.

##### 3.7.2.1. Limit of quantification and detection (LOQ and LOD)

ISD and HDZ LOQ and LOD values were calculated using a signal-to-noise ratio approach in accordance with ICH guidelines, and practically executed LOQ and LOQ solutions in the UPLC.

#### 3.7.3. Accuracy

The consistency of an analytical technique refers to how similar the method's results are to the actual value. As seen in Table 3, the accuracy results showed the percentage recovery in the range of 98.4–101.9% at all three levels (80, 100, and 120%). The percentage recovery findings were within the acceptable level, and ranging from 98.0% to 102.0%, indicating that the procedure should be used for routine drug evaluation.

**Table:** Accuracy and precision data for ISD and HDZ

Drug	Amount	Amt found	% Recovery	Mean	Repeatability % RSD
ISD	20	20.32	101.6	100.85 ± 0.901	0.893
	20	19.97	99.85		
	20	20.22	101.1		
	40	40.37	100.93	101.27 ± 0.507	0.501
	40	40.74	101.85		
	40	40.41	101.03		
	60	60.96	101.6	99.89 ± 1.638	1.6407
	60	59.84	99.73		
	60	59	98.33		
HDZ	37.5	37.8	100.8	100.54 ± 1.444	1.436
	37.5	38.19	101.84		
	37.5	37.12	98.99		
	75	75.66	100.88	100.52 ± 1.046	1.0408
	75	74.51	99.35		
	75	76.01	101.35		
	112.5	112.67	100.15	99.40 ± 0.992	0.998
	112.5	112.25	99.78		
	112.5	110.56	98.28		

#### 3.7.4. Precision

The relative standard deviation (RSD) is used to express the precision of a system, which is well-defined as “the closest of agreement between a set of measurements obtained from multiple samplings of the same given criterion under the specified conditions.”

The system, intermediate, and method precision results indicated that the method is accurate within reasonable limits for both solutions. The tailing factor, % RSD, and a number of theoretical plats were calculated; all of the outcomes were within acceptable limits. Table 3 shows that the acceptable precision for the RSD was less than 2.0%.

#### 3.7.5. Solution stability

The consistency of the mobile phase, standard, and sample solutions was evaluated by maintaining the solutions in different storing conditions, which was 15 days for the mobile phase and four days for the standard and sample solutions, and monitoring for variations in both intensity and retention of the peaks. Additionally, the standard and test solutions were monitored at both refrigerated and room temperature for the specified times, and the % difference was measured and evaluated using a chromatogram of freshly prepared solutions. The relative standard deviation (%) between ISD and HDZ was determined using the original conditions' values, and should be less than 2.0%. The results indicate that the standard solution was stable at 25 °C for 30 h, seven days at room temperature, and 15 days when refrigerated. The study solution remained relatively stable at 25 °C for 30 h during the assay, while the mobile phase remained stable for 30 days in ambient conditions.

### 3.8. Forced degradation of ISD and HDZ

**Fig. 9:**
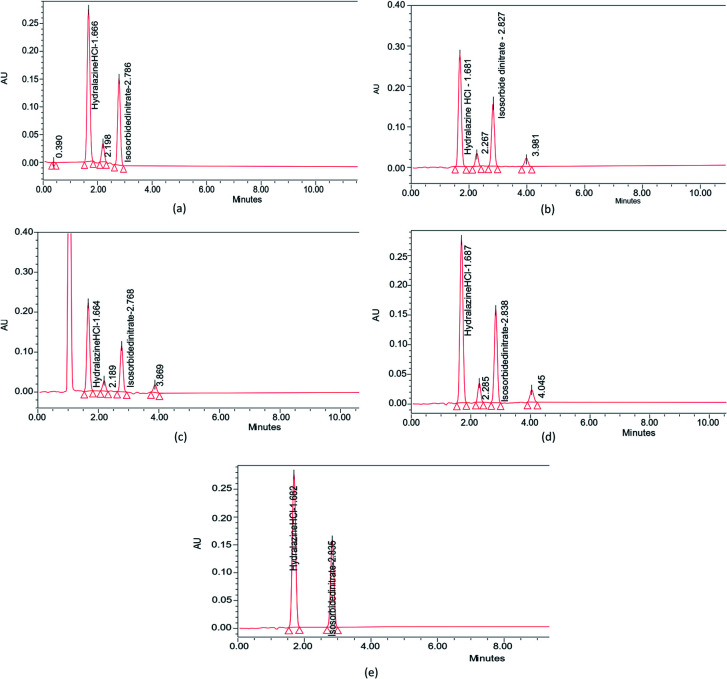
Forced degradation studies of ISD and HDZ in (a) 0.1 M HCl, (b) 0.1 M NaOH, (c) 3% H_2_O_2_, (d) photodegradation in UV light, and (e) control without any stress.

Forced degradation tests were undertaken on the ISD and HDZ drug combinations. The 0.1 M NaOH, 0.1 M HCl, 0.3% H_2_O_2_, and UV light were picked as a primary stress condition. This gave reliable information regarding the stability of ISD and HDZ. No drug was degraded instantly after adding the acid, base, or peroxide, allowing it to stand for 1, 3, and 6 h. The study report after 1 and 3 h has not shown a noticeable degradation, and thus the samples were allowed to stand for 6 h. It was found that the drugs tend to degrade at this time, and the results and the degradation peaks are portrayed in Table 4 and Fig. 9.

**Table:** Forced degradation studies for ISD and HDZ

Conditions	Drug area		% Drug recovery		% Drug degradation	
	ISD	HDZ	ISD	HDZ	ISD	HDZ
Acid	1 251 843	2 234 263	99.434	86.462	0.5654	13.53
Alkali	1 087 853	2 185 874	86.408	84.587	13.591	15.412
Peroxide	1 042 272	2 125 988	82.788	82.270	17.21	17.729
Photo	1 148 252	2 217 951	91.206	85.828	8.793	14.171
Control	1 258 962	2 584 152	100	100	0	0

### 3.9. Assay of marketed formulation

As shown in Table 5, the test results indicated an optimum percentage of ISD and HDZ in the Isolazine tablets. This demonstrates the selectivity of the technique for determining ISD and HDZ in tablets, where the results were good and achieved without any detected interference from the excipients. Additionally, the result was compared to the developed HPLC technique using the student *t*- and *F*-tests, and was determined to have the best results. The suggested approach is successfully used to determine the presence of ISD and HDZ in the tablets.

Statistical analysis of the proposed and reported method comparison

**Table:** 

	*t*-Test: two-sample assuming unequal variances			
	ISD		HDZ	
	Variable 1	Variable 2	Variable 1	Variable 2
Mean	99.70666	99.1733	99.91	99.053
Variance	0.1132	0.02003	0.0997	0.0305
Observations	3	3	3	3
Hypothesized mean difference	0		0	
d*f*	3		3	
*t* stat	2.5304		4.1116	
*P*(*T* ≤ *t*) one-tail	0.04269		0.01302	
*t* critical one-tail	2.35336		2.35336	
*P*(*T* ≤ *t*) two-tail	0.08539		0.02605	
*t* critical two-tail	3.18244		3.1824

**Table:** 

*F*-Test two-sample for variances				
Mean	99.70666667	99.173	99.91	99.0533
Variance	0.113233333	0.02003	0.0997	0.03053
Observations	3	3	3	3
d*f*	2	2	2	2
*F*	5.6522		3.2652	
*P*(*F* ≤ *f*) one-tail	0.15032		0.23445	
*F* critical one-tail	19		19	

### 3.10. Wastage recycling

**Fig. 10:**
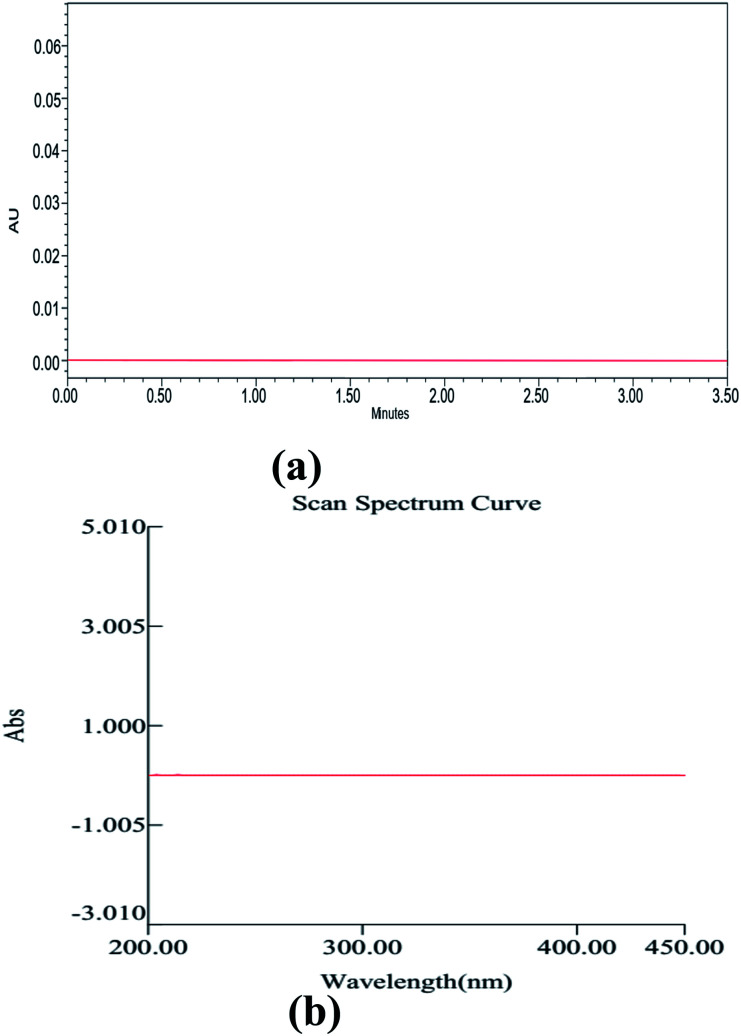
UPLC chromatogram (a) and UV spectrum of ethanol after distillation using HPLC grade ethanol as a blank.

Method development and validation have utilized an enormous amount of solvent and generated waste, approximately 500 mL, including the mobile phase for analysis, washing the column, and solvents used for other purposes of analysis. Among this, the major part of the generated waste is around 70% ethanol, 0.1% TFA, and remaining water. So, we aimed to recycle this wasted ethanol by using a simple distillation process and regenerated the waste, and helped Mother Earth from further pollution. Although ethanol falls under the category of a bio-degradable solvent, wastage recycling is a fundamental principle in the GAC principles. The distilled ethanol was further cross-checked for its purity by using the UV spectrophotometry across the UV region (200–400 nm) using HPLC grade ethanol as a blank (Fig. 10). With the help of UPLC, we also found no impurities in the distilled ethanol, which was further marked and utilized for the analysis of drugs.

### 3.11. Green assessment for the developed method

The method combines tri-combinations for the analysis of two drugs. Among these three, every aspect has its equal importance in method development. In this contest, a developed method simply cannot claim it as eco-friendly without evaluation using proper assessment tools. Here, the method was evaluated by using 5 assessment tools called the National Environmental Methods Index (NEMI), Eco-scale Assessment (ESA), Green Analytical Procedure Index (GAPI), Analytical Method Greenness Score (AMGS), and Analytical GREEnness Metric (AGREE), and assessed the method greenness. Each tool has its own set of benefits, drawbacks, and evaluation process. The final data acquired from each evaluation tool may lead to a different conclusion on which approach is the greenest, and choosing which evaluation technique to use. However, although different tools were used in this method assessment process, all of the tools showed their results in a single path that is eco-friendly and greenest. The evaluation of the method was done as follows:

NEMI is a classical qualitative assessment tool developed for evaluating green chemistry. Initially, it is the only tool used for the evaluation of the GAC methods. Although new assessment tools were developed for the GAC, NEMI has its advantages in assessing the green analytical method. NEMI is represented by a color-coding (green and colorless) circular pictogram, which is divided into four quadrants. Each quadrant has its own assessing criteria, like quadrant one deals with the list of chemicals issued by the Environmental Protection Agency's (EPA) Toxic Regulatory Inventory^25^ (TRI) list of Persistent Bioaccumulative Toxic (PBT) chemicals. The chemicals used in this method have not been listed in PBT, so this quadrant is coded as a green color. The second quadrant deals with hazardous chemicals, which are under the Resource Conservation and Recovery Act^26^ (RCRA) by EPA.

**Fig. 11:**
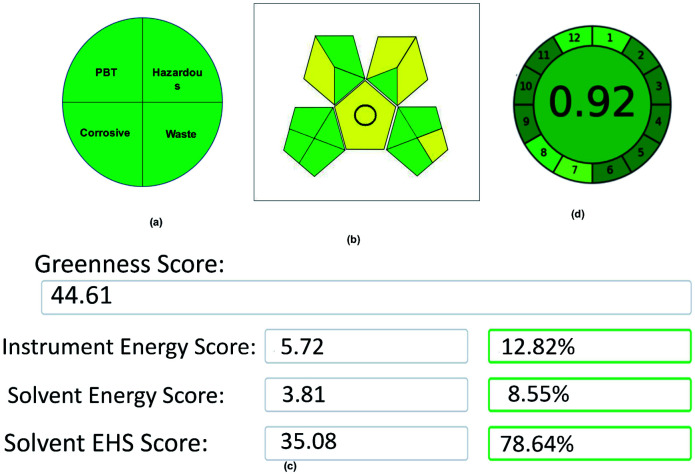
Green assessment results for the proposed method: (a) NEMI, (b) GAPI, (c) AMGS, and (d) AGREE metrics.

In the same way, the chemicals listed in the RCRA list were present in this method, so the second quadrant is coded with a green color. The third quadrant deals with the pH of the solutions used for analysis, which should be between 2 and 12 to make this quadrant green, and the mobile phase pH used in the method was under the range, so the third quadrant was coded as a green color. Finally, the fourth quadrant deals with wastage, as the total wastage should be less than 50 g or mL. In this method, the wastage is negligible due to incorporating the recycling procedure, so the third quadrant was coded as a green color. The overall NEMI pictogram of the method is depicted in Fig. 11.

#### 3.11.1. GAPI

GAPI is a modified version of NEMI, which consists of 11 subdivisions and is coded with red, yellow, and green, indicating danger, moderate and eco-friendly, respectively. Assessment by using GAPI was made simplified by J. Płotka-Wasylka^27,28^ in his article by developing free software. Application of the method details that need to be assessed has to apply in the software, which consists of 11 simple steps to generate the result. The generated result was depicted in Fig. 11, which shows the method's sustainability and its future scope.

#### 3.11.2. AES

AES^29^ is another assessment tool that deals with the penalty points (PP) scored by the method. PP is calculated based on four major divisions as reagents or chemicals PP scored, the instrument and amount of energy used, the amount of waste generated by the total method, and the occupational hazard for an analyst. After calculating the overall penalty points from the above four steps, AES is calculated as AES = 100 − PP. The PP's generated by this advanced method was four, and the final AES score is 96, showing the method's efficiency towards its sustainability. The overall calculation of AES is depicted in Table 6.

**Table:** Detailed calculation of AES for the proposed method

Solvent energy calculation				
Solvents	Pictogram	GSH signal word	Amount of solvent	subtotal
Ethanol	2	Danger	10–100 mL	4
TFA	0	0	1	0
Water	0	0	0	0
Instrumental PP calculation				
Energy	For UPLC ≤ 0.1 kW h per sample			0
Occupational hazard (OH)	No OH due to use of bio solvent and hermitization process			0
Wastage and recycling	0–10 mL			0
			Total PP	4
			AES	96

#### 3.11.3. AMGS

AMGS^30^ is another assessment tool used for the assessment, a combined and modified version of the HPLC environmental assessment tool (HPLC-EAT) and Safety Health and Environmental assessment (SHE). Overall, the AMGS results were divided into three categories: an instrument, solvent energy, and solvent Environmental Health and Safety (EHS) score. The overall score of the method is based on the aggregation of these three scores, where it should be as low as possible to make the method greenest. After feeding the required data in the spreadsheet provided by the AES Green Chemistry Institute for the green assessment, the final score obtained for the proposed method was 44.61 (Fig. 11), which shows the positive impact of the developed method on the environment.

#### 3.11.4. AGREE metrics

AGREE metrics^31,32^ is the latest green assessment tool that covers all 12 green analytical principles. The overall result was based on the individual principles score obtained from the individual principles, and represented as 1. A value closer to one indicates the method greenness. After feeding the method details in the software, the overall result shows (Fig. 11) the effect of the method on the environment as very friendly and sustainable for a longer time.

Although five assessment tools used different strategies or procedures for assessing the method greenness, the overall goal was to determine the method's sustainability. All of the methods, irrespective of their strategies, suggested that this method is eco-friendly and adaptable for future green analysis without any hurdles by their results.

## 4. Conclusion

The study's primary objective was to develop a GAC-based stability-indicating RP-UPLC technique to detect ISD and HDZ in the bulk and pharmaceutical dosage forms. To achieve this, AQbD was applied to develop a method that can be used for long-term utility without further revalidation. By the MODR, a resilient method area was proposed with a flow rate at 0.45–0.55 mL min^−1^, column temperature at 32–38 °C, and ethanol variation of 5%. By the mathematical model, we acquired a deeper grasp of the implications of the method parameters on the results. When executed, the developed method found the shortest runtime of 3.5 min and good resolution between the two drugs by 5.4. The degradation peaks generated after forced degradation studies also show a better resolution, and separation of ISD and HDZ from the degradation products using the proposed LC technique. The devised analytical technique was verified in terms of the reproducibility, accuracy, sensibility, and linearity at the designated working point, and found to be within limits. Finally, the method was validated using five green assessment tools called NEMI, GAPI, Analytical Eco-Scale (96), AMGS (44.61), and AGREE metrics (0.92), and found to have the best eco-friendly results. The application of waste recycling makes this method stands alone due to zero wastage of the organic phase, and helps the environment from further damage. This method shall also help the quality control departments in the industries and commercial labs in adopting and analyzing these combinations in the bulk and tablet dosage forms. The future prospects of this study may dominate the scientific community to adopt and develop such eco-friendly robust AQbD-based methods for analyzing various chemical substances using green solvents.

## Conflicts of interest

There are no conflicts to declare.

## Supplementary Material
